# Patterns in PARTNERing across Public Health Collaboratives

**DOI:** 10.3390/ijerph121012412

**Published:** 2015-10-05

**Authors:** Christine A. Bevc, Jessica H. Retrum, Danielle M. Varda

**Affiliations:** 1 North Carolina Institute for Public Health, University of North Carolina, Chapel Hill, Campus Box # 8165, Chapel Hill, NC 27599, USA; 2 Department of Social Work, Metropolitan State University of Denver, Campus Box 70, P.O. Box 173362, Denver, CO 80217-3362, USA; E-Mail: jhaxton1@msudenver.edu; 3 School of Public Affairs, University of Colorado-Denver, 1380 Lawrence St., Ste. 500, Denver, CO 80204, USA; E-Mail: Danielle.Varda@ucdenver.edu

**Keywords:** social network analysis, public health collaboratives, heterophily, partnerships

## Abstract

Inter-organizational networks represent one of the most promising practice-based approaches in public health as a way to attain resources, share knowledge, and, in turn, improve population health outcomes. However, the interdependencies and effectiveness related to the structure, management, and costs of these networks represents a critical item to be addressed. The objective of this research is to identify and determine the extent to which potential partnering patterns influence the structure of collaborative networks. This study examines data collected by PARTNER, specifically public health networks (*n* = 162), to better understand the structured relationships and interactions among public health organizations and their partners, in relation to collaborative activities. Combined with descriptive analysis, we focus on the composition of public health collaboratives in a series of Exponential Random Graph (ERG) models to examine the partnerships between different organization types to identify the attribute-based effects promoting the formation of network ties within and across collaboratives. We found high variation within and between these collaboratives including composition, diversity, and interactions. The findings of this research suggest common and frequent types of partnerships, as well as opportunities to develop new collaborations. The result of this analysis offer additional evidence to inform and strengthen public health practice partnerships.

## 1. Introduction

There is a growing recognition that traditional hierarchical structures have become increasingly problematic and inefficient at promoting collaborative and innovative partnerships within and across the public and private sectors. Recent public health policy and program guidance has increasingly encouraged and promoted collaborative structures and partnerships that strongly support a network approach to governance. When confronted with challenges, organizations do not work alone. Inter-organizational networks represent one of the most promising practice-based approaches in public health as a way to attain resources, share knowledge, and, in turn, improve population health outcomes. As a result of macro-level social changes, the structure of political systems themselves are being transformed from hierarchically organized, unitary systems of government to more horizontally organized and relatively fragmented systems of governance, in which government plays a far less central role in terms of direct control and administrative arrangements [1,2,3,4,5,6,7].

The power of networks in helping to understand issues of administration and management extends beyond the realm of public health. The results of this study identify factors that are also significant with regard to issues of business continuity, organizational resilience, resource dependencies, organizational effectiveness, and the potential liability of unconnectedness. In the post-9/11, post-Katrina (and post-Sandy) political environment, there has been a continued emphasis on “connecting the dots” and bridging the gap across the various professional disciplines. Government policies and programs have implemented requirements for intergovernmental cooperation and collaboration with the intent that networks would increase response capabilities and improve the overall communication among organizations.

Researchers and practitioners have begun to implement policies that encourage organizations to work together. Policies not only promote innovation and creativity, they also stimulate competition in the private sector [8]. The consequences of not collaborating meant organizations might not succeed or, worse, might fail in their management [8]. While collaboration among government agencies is certainly not new to public health, formalization of these practices into policy represents a shift in these bureaucratic structures’ traditional myopia. Combined, interorganizational networks and collaborative arrangements may, in fact, increase efficiency and effectiveness in confronting complex public health and policy-related problems by leveraging resources and relationships. However, the resource intensive costs related to managing these networks and how to ensure networks are effective and efficient represents critical items on the Public Health Systems and Services Research (PHSSR) research agenda. With the growing importance of network governance, the necessity to test and understand the structure of networks, and, subsequently, determinants of interaction within them, has become increasingly evident.

In this study, we apply a systems science approach to help identify and, ultimately, inform stakeholders involved in inter-organizational networks, including a range of public, private, and nonprofit partners. The objective of this research investigation is to identify and determine the extent to which potential partnering patterns influence the structure of networks. While the literature on the importance of a network approach has grown, research demonstrating the most effective practices has not kept up with the pace of implementation in practice. Listed as one of the Ten Essential Public Health Services, inter-organizational networks, referred to herein as public health collaboratives (PHCs), represent an essential function of public health agencies. Public health now includes traditional health partners, “but also entities that operate outside the traditional sphere of healthcare, such as faith-based and other non-health community-based organizations, schools, businesses, and other non-health governmental agencies…PCHs are frequently established to leverage resources and maximize the synergies that many agencies bring to the table” [9] (p. E1) as “networks” of organizations working collaboratively [10]. Given the increasing use of networks as an approach in public health, evidence to inform the management and evaluation of effective networks is essential. The results of this analysis offer additional evidence to inform and strengthen public health practice partnerships.

## 2. Experimental Section

Networks offer a much more holistic approach to addressing community needs, in contrast to a more individualistic application. When organizations act in a vacuum (not as part of a network), there are risks resulting in inefficient use of resources. For example, actions and objectives of one organization may not align or even conflict with the goals and interactions of other organizations targeting the same population or there may be unnecessary redundancy in services, while leaving gaps in others. However, traditional research methods operationalizing organizations or programs as the unit of analysis are inadequate for examining network behavior. Social network analysis [11] is used to identify and compare network features and mechanisms (*i.e.* who is connected to whom and the quality of those relationships) within each collaborative. This study examines the combination of attribute and relational data collected by PARTNER, a social network analysis tool designed for practitioners, funded by Robert Wood Johnson Foundation, to better understand the structured relationships and interactions among public health organizations and their partners, in relation to collaborative activities. Combined with descriptive analysis, we address potential partner preferences across the networks. Specifically, we use a series of *exponential random graph* (ERG) models applied to the set of public health collaborative (PHCs) networks to: (1) examine partnerships between different organization types; (2) understand the propensity of organizations to form of partnerships with certain organizations based on their attributes; and (3) identify frequency and significance of partnership within and across collaboratives.

As previous research has demonstrated, activities undertaken by public health collaboratives may be influenced, though limited in extent, by “disciplinary silos,” a pattern that would partly support the argument that “similarity breeds connection” [12,13,14,15,16,17]. Here, it is also important to consider interdependencies and the potential for alliance formation between dissimilar organizations, called anti-homophily. This pattern reflects the tendency of dissimilar organizations to select one another, also referred to as disassortative mixing [18], or heterophily. This research addressed the question, to what extent do organizations in public health collaboratives form alliances, or partnerships, with various types of organizations?

### 2.1. Data Sets and Sources

We use data from the *Program to Analyze, Record, and Track Networks to Enhance Relationships* (PARTNER) Tool to understand organizational actions within the context of structured relationships and, subsequently, the network structures themselves. PARTNER is an online social network data collection and analysis tool designed to measure and monitor collaboration among members of varying networks, which has resulted in an unprecedented and robust network dataset of public health interorganizational networks [9]. PARTNER is designed for and used primarily by practitioners who are engaged in interorganizational networks in their communities, often as a way to evaluate or assess their collaborative processes and outcomes. Although each community self-identifies the members of their networks and the topic of the work they do together, to be included in this analysis, they had to be (1) interorganizational and (2) public health focused. Through the use of a consistent data collection methodology and common set of core questions, the PARTNER metadata set enables us to more effectively explore potential partner preference across multiple public health collaboratives (PHCs) [9,19,20,21]. Using a survey and roster-based instrument, organizations subsequently answer attribute and relational questions about their organization, as well as how frequently they interact with each organization, the quality of those interactions, and perceptions of trust and organizational value for each partner organization (using three measures of trust and three measures of value).

For the purposes of this analysis, we focus on the attribute of organization type and the presence (or absence) of reported interactions among organizations in each public health collaborative. The dataset for this study contains a subset of 162 whole networks consisting of approximately 4500 organizations; and 18,000 interactions, defined as the presence of a directed tie (where x*_ij_* = 1) between two organizations (dyad) [22]. As previous research discussed, PHCs vary in terms of size and focus; however, PARTNER networks in this study were selected based on their high response rate threshold (>70%), composition of a bounded group of organizations within a community, and collaborative goal to address a public health topic [17].

### 2.2. Analysis

This study analyzes the PARTNER PHC networks using descriptive quantitative and social network analysis methods. This analysis examines the predictors of dyadic interaction based on fifteen (15) organizational types to determine the extent to which organizations work with similar (the same organization types) *versus* dissimilar (different organizations types) alters (e.g., public health working with nonprofits, or non-public health government agencies working with private businesses). The organization types include public health, education, funders, medical care, government (non-public health), non-profits, health insurance, professional organizations, faith-based organizations, business, law enforcement/legal, military, regional networks/alliances, citizen representatives/advocates/experts, and community health centers. Data are analyzed on the network level to identify mechanisms promoting (or deterring) the formation of network ties, assessing the influence of exogenous, independent organizational attribute effects within each network.

**Figure 1:**
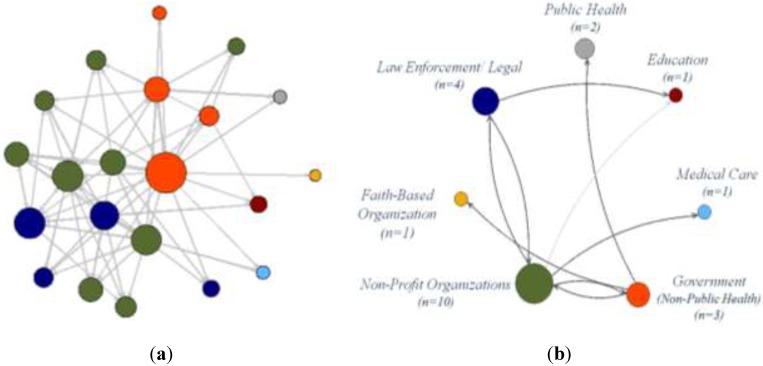
(**a**) Traditional network graph of interactions within a public health collaborative (PHC). (**b**) Reduced network graph of inter-organizational interactions.

The approach for this analysis follows Snijders and Baerveldt [23], where we employ a multilevel approach to examine the patterns in partner relations across PHCs. In this two-step approach, “the micro level is the study of the relational ties within each single network, and the macro level is the combination of these multiple networks studies” [23] (p. 124). Here, we first identify the parameter estimates and standard errors for each PHC and, then, summarize these estimates across the networks, which allows us to more closely examine the predictors of dyadic interactions. The extension of multilevel analysis to relational data draws from methods previously proposed by a series of researchers [24,25,26,27,28]. Using the “nodemix” term [29], we use the ERG models to test whether one organization type works with each of the other organization types in the PHC; controlling for these terms helps to control for patterns of “mixing” for categorical attributes [29]. As an example, Figure 1a depicts the interactions within a single public health collaborative; differentiating between the seven different organization types represented in the collaborative’s membership. Figure 1b offers a reduced illustration of these interactions, showing the presence and frequency of partnerships between the different types.

In this PHC, the greatest numbers of interactions were found between the non-profit organizations (*n* = 10), government (*n* = 3), law enforcement/legal (*n* = 4), and education (*n* = 1), who interacted with two or more organization types. Public health (*n* = 2), medical care (*n* = 1), and faith-based organizations (*n* = 1) interacted with only one other type of organization. In the network analysis, the ERG model tests whether these observed interactions between organization types are significantly more (or less) than expected, based on an equal probability of 0.5 between all types. This will allow us to determine, for example, whether local health departments are more likely (or not) to partner with non-profits, faith-based organizations, or any other organization type, in their network, informing the degree to which the public health sector remains siloed or not. Each combination of the same model is repeated for each of the 162 PHCs used to test each of the PHCs individually and the results are aggregated to summarize the interaction behaviors among organizations across all PHCs.

All analysis was conducted in R with the assistance of the *statnet* package [30].

## 3. Results and Discussion

Across the 162 PHCs, we found high variation and significant differences within and between these collaboratives including composition, diversity, and interactions. Membership of the PHCs ranged from 7 to 99 (μ = 18), with collaboratives containing four types of organizations, on average. However, diversity did vary within each PHC from 1 to 10 organization types. As previous research found, the diversity of PHC membership was positively correlated with the size of the collaborative [17]. Members reported a wide range of relationships with other organizations, averaging a reported 9 to 10 partners. The findings provide insight as to whether frequent and/or significant dyadic patterns emerge within these partnerships.

### 3.1. Frequency of Partnerships

The first step of the analysis examined the frequency of partnerships within and across the 162 PHCs. More specifically, the presence or absence of a tie between two organization types, as well as the total number of times these partnerships occurred. The overall frequency among the 225 possible dyadic (paired) combinations was used to rank partnerships from most common to least frequent. Table 1 provides a summary of partnership frequency observed across all 162 PHCs, where the cells represent the number of networks that contain a reported interaction between the two organization types. As noted previously, the composition and diversity of these networks varies considerably [17], the last column presents the total number of PHCs with one or more of the organization types in their network. The diagonal of the table represents partnerships between the same organizations, e.g., public health and public health, whereby the network must contain two or more organizations of the same type. For example, although there are 162 networks that contain at least one public health organization (criteria for a network to be included in this analysis); however, there are only 123 in which we found interactions reported between two public health organizations. The most frequent partnerships were found to occur between non-profit organizations and other organization types, including government, public health, education, medical care, faith-based organizations, law enforcement, and professional organizations. Other frequently identified partnerships included those between medical care and public health, business and government, and citizen advocacy groups and public health.

In contrast, the least common partnerships across the PHCs were among funders, health insurance organizations, and military organizations, which we suspect is due largely to their overall low membership for these types across the PHCs. Even with this low membership, funders were more likely to report engaging with other organizations than vice versa, with a few exceptions where there were no reported partnerships by either type, including military entities and community health centers. Health insurance organizations were similar; however, they interacted with even fewer types of organizations, lacking connections with law enforcement/legal organizations, military, regional networks/alliances, and community health centers. Although military involvement was limited, there was engagement with all other types of organizations, except funders.

**Table 1:** Frequency of partnerships between organization types across PARTNER PHCs (*n* = 162).

Organization Type		(1)	(2)	(3)	(4)	(5)	(6)	(7)	(8)	(9)	(10)	(11)	(12)	(13)	(14)	(15)	Total Organizations
(1)	Public Health	123															162
(2)	Education	84	102														113
(3)	Funders	4	2	7													11
(4)	Medical Care	67	57	4	82												87
(5)	Government (Non-Public Health)	92	85	4	65	115											121
(6)	Non-Profits	101	87	5	65	105	127										130
(7)	Health Insurance	6	5	2	5	7	6	8									9
(8)	Professional Organizations	13	9	1	8	12	14	1	14								19
(9)	Faith-Based Organizations	31	27	2	26	32	33	0	4	34							37
(10)	Business	37	41	2	30	45	43	5	7	15	47						49
(11)	Law Enforcement/Legal	25	26	1	22	27	29	0	5	17	16	32					34
(12)	Military	7	6	0	7	6	8	0	1	4	4	4	8				8
(13)	Regional Networks/Alliances	10	9	1	7	10	10	0	1	7	6	5	1	10			12
(14)	Citizen Representatives/Advocates	33	22	2	22	24	25	1	1	6	10	4	1	3	39		49
(15)	Community Health Center	12	12	0	12	13	14	0	4	6	6	4	4	3	4	14	19

**Figure 2:**
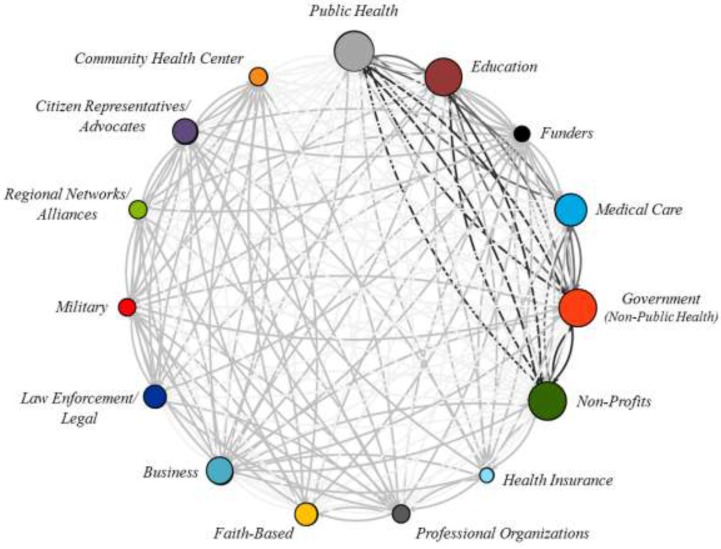
Frequency of partnerships across all public health collaboratives.

To help summarize these results, Figure 2 (below) provides a summary of the interactions between the fifteen organization types, using a network graph circle projection, whereby nodes are placed clockwise in a circle based on the frequency of partnerships. In this figure, the size of the node is weighted by organizational frequency and the edges (connecting lines) are shaded based on the calculated *z*-scores, or standard deviation from the mean frequency of dyadic partnerships (μ = 20, σ = 27), where the darker lines represent greater positive deviation in the partnership frequency between the two organization types across PHCs.

Given the public health focus of these collaboratives, partnerships with public health agencies varied, as illustrated in Figure 2. Public health agencies were found to most commonly work with non-profits (101 out of 127 PHCs), government agencies (92 out of 115 PHCs), education (84 out of 102 PHCs), medical care (67 out of 82 PHCs), and businesses (37 out of 47 PHCs). Public health agencies were least likely to develop partnerships with funders (4 out of 7), health insurance (6 out of 8 PHCs), military (7 out of 8 PHCs), and regional networks (12 out of 14 PHCs). The findings of this research suggest common and frequent types of partnerships (most commonly with non-profit organizations), as well as the less frequent interactions with financial organizations, who had lower levels of participation in PHCs, overall.

### 3.2. ERGM Results

The calculation of standard deviations and analysis of frequency relies on the assumption of the statistical independence of dyadic partnerships. However, to account for the inherent dependence of partnership patterns within these collaborative networks, the second step of our analysis tests whether these partnerships are, in fact, statistically occurring more frequently relative to the others within each network. The ERG model allows us to incorporate the type of organization in a probabilistic model to determine whether there are any preferential partnerships or biases toward partnering with a specific type of organization in a PHC. Ideally, the lack of significant finding indicates an equal likelihood to form partnerships, where the potential bias is low. In other words, public health organizations are statistically no more likely to work with non-profits than any other organization type; organizations are found to thereby have an equal opportunity (statistically) to interact and develop partnerships with other organizations in the network.

Summarizing all 162 networks, the combined results of the ERG models identified *very few* (6.2%, *n*_PHC_ = 10 out of 162 PHCs) networks with statistically significant biases in partnerships between one or more organization types within networks. More often, partner preferences were limited between only a couple organizations in the network, *i.e.* government agencies and public health departments. Among these ten networks, their size varied from 6 to 30, with a range of 1 to 11 significant partnerships in each PHC (μ = 2). These significant partnerships were found between a variety of organization types, with no particular partnership occurring more than three times in the networks. Reflecting upon our research question, the extent to which organizations partner with other types of organizations varies considerably; however, they share a relatively equal probability of partnering with these other types.

While not all possible combinations were present in these networks, the ERG model results and the absence of these partnerships does not suggest that the lack of interactions is owing to any preferential bias happening in organizations partnership choices. It should also be noted that we also base our analysis on the assumption that the organizational composition of each PHC is mutually exclusive. Taking these assumptions into consideration, the results strongly suggest that organizations in these networks are just as likely to form partnerships with one type *versus* another.

### 3.3. Discussion

Our findings reveal several important contributions to public health practice related to inter-organizational networks. Because we have an unprecedented number of PHC’s in one data based (most studies are only able to examine one or a few at a time) we were able to look at a very large and broad sampling of PHCs from across the U.S. to examine partnership behavior within networks in a way that has not been done before. While early studies suggested that centralized “star” networks are most efficient in decision-making and problem solving [31,32], this centralization simultaneously creates vulnerability in the network, potentially allowing decision-makers to be overwhelmed by information and problems [32]. As an alternative to these centralized and hierarchical systems, decentralized, collaborative networks are considered as a more democratic means of developing public policy and administration [8,33,34,35]. As displayed in the visualizations in this paper, a common trait across all 162 networks analyzed in this study is the interconnected, decentralized structure of the networks. This pattern corresponds to the literature suggesting that centralized, top-down approaches are no longer effective in meeting complex public health (and other social issues) in communities. These networks and the growing body of literature on the topic support their application to overcome challenges facing contemporary public management and goals [36,37,38,39,40,41]. However, managing networks does not come without challenges and does require “network leaders” (the person tasked to manage and facilitate PHCs) to develop skills that navigate issues of accountability, autonomy, and organizational culture and mission differences [10].

Not only is the decentralized structure of these networks notable, but the diversity of membership in them reflects the growing practice to work across boundaries. The findings of this research suggest common and frequent types of partnerships (e.g., with nonprofit organizations), as well as the potential to develop opportunities and new collaborations with financial organizations, who had lower levels of participation in PHCs, overall. Identifying patterns of common partnerships (here, with nonprofit organizations) and less common partnerships (e.g., with financial organizations) allows us to better understand where the majority of resources are shared across the public health system. The frequent partnerships with government and education agencies in these PHCs is not surprising, as public health and education agencies are often themselves government agencies. A predominant network theory is that of homophily, asserting that organizations with similar characteristics will have a preference to form ties. While these organizations were coded separately in the data, education, public health, and government agencies are most often partnering with public agencies and it is not uncommon for public agencies to prefer to form ties (given their common organizational cultures and characteristics). So while we did not find a significant preference for any one type of partnership, if we were to collapse these groups into one category, we may find a stronger relationship. Regardless, findings provide strong evidence that organizations in PHCs are forming ties across diverse types which is presumably leading to a greater range of resources, knowledge, and information sharing—all characteristics thought to improve the chances of effective outcomes. Additional research is needed to explore whether there are additional mechanisms influencing the topology of these networks, including alternative exogenous or endogenous factors.

Some limitations of this study relate to the lack of access to specific demographic characteristics of PHCs such as geographic scope or socioeconomic information about the communities these PHCs serve. For example, there could be potential differences between organizational partnering behavior for rural areas rather than those in urban, as has been found in a study of local health departments [42]. Even though this analysis was done on the largest collection of secondary data on public health partnership related networks to date, networks who are interested in evaluating their network activities are the ones who use PARTNER, *i.e.*, those who would be self-selected into this sample. Therefore, it is possible that this sample contains networks that are unique, perhaps more high performing networks.

## 4. Conclusions

The preliminary findings of this research suggest common and frequent types of partnerships, as well as opportunities to develop new collaborations. By assessing networks in a reduced approach, we can simplify the complexity of these relationships in a more interpretable manner. This allows us to see which organization types are more prevalent and active in PHCs. This enables us to better understand where organizations may tend to form preferential partnerships, or not. In these data, we do not see evidence of these possible preferential biases. Although there seems to be a more frequent type of partnership (PH-Nonprofit), this partnership is not significantly different (statistically) from other types of partnerships. In these models, we address the basic presence or absence of partnerships. Future research would be well-suited to more closely evaluate whether preferential tendencies may emerge within network when considering the variation in the types of relational ties, thereby discerning whether internal alliances may develop as a result of varying measures of interdependency.

These findings inform practice in several ways. First, for practitioners who participate in public health partnership networks, the biggest implications of this study are to consider using systems focused network leadership strategies to continue nurturing productive partnerships and to facilitate partnerships among unlikely organizations (e.g., between funding organizations and non-profits whose network resources would be stronger if this partnership existed). Without a strategy and network leadership skills, practitioners may not have a way to manage their partnerships except with a “more partnerships is better” strategy, leading to redundancy and the potential to overwhelm an already resource-scarce system [20]. Second, no formal definition of the “public health system” is currently available. While this research does not provide such a definition, it does tell us more about the “who” (is a part of the system) and “what” (the systems partners prefer as partnerships) of public health systems. As the “culture of health” continues to emphasize cross-sectoral partnerships as the foundation, these kinds of findings begin to tell us what that landscape currently looks like.
